# Neurological Complications Associated With COVID-19 Compared to Other Viral Infections: A Systematic Review of Current Evidence

**DOI:** 10.7759/cureus.101817

**Published:** 2026-01-18

**Authors:** Ifeoluwa s Bakare, Victor O Olaiya, Olurotimi J Badero, Chukwumbana Faith Okirie

**Affiliations:** 1 Department of General Medicine, Ternopil State Medical University, Ternopil, UKR; 2 Department of Cardiology, Izhevsk State Medical Academy, Izhevsk, RUS; 3 Department of Interventional Cardiology, Iwosan Lagoon Hospital, Lagos, NGA; 4 Department of Interventional Cardiology, Division of Cardio-Nephrology, Cardiac Renal & Vascular Associates, Jackson, USA; 5 Faculty of Medicine, Near East University, Nicosia, Cyprus

**Keywords:** dengue manifestation, influenza b, neuroinflammation, neurological complications with covid-19, sars-cov-2

## Abstract

Neurological complications have become one of the most concerning features of COVID-19, yet clinicians still lack a clear comparison between these findings and what is seen in other viral infections. Understanding where SARS-CoV-2 fits, whether it behaves like influenza and dengue or follows an entirely different pattern, is essential for diagnosis, management, and planning long-term care. We conducted a systematic review following Preferred Reporting Items for Systematic Reviews and Meta-Analyses (PRISMA) guidelines (PROSPERO: CRD420251064831). Searches across PubMed, Scopus, and Web of Science (2000-2025) identified 24 eligible studies, including observational cohorts, clinical trials, case series, autopsy work, and national surveillance data. Because of the wide variation in study design and reporting, a narrative synthesis was used.

Across the 24 studies, COVID-19 exhibited the widest and most severe spectrum of neurological involvement. Reported central nervous system complications included ischemic stroke, encephalopathy or encephalitis, seizures, and extensive microglial and white-matter injury in fatal cases. Peripheral complications were also prominent, such as anosmia, demyelinating neuropathies, Guillain-Barré syndrome (GBS), chronic inflammatory demyelinating polyneuropathy, functional movement disorders, and persistent abnormalities on nerve conduction testing long after recovery. In contrast, neurological complications from influenza were less frequent and mostly involved encephalitis/encephalopathy, seizures, meningitis, GBS, or myelitis, with generally low mortality. Dengue virus has been associated with a spectrum of direct neurotropic effects and immune-mediated syndromes, including encephalitis, GBS, myelitis, brachial neuritis, and myositis. Most patients recovered, and mortality remained low. Compared with influenza and dengue, COVID-19 stands out for both the breadth and severity of its neurological manifestations, as well as the persistence of symptoms in many survivors. These findings highlight the need for early neurological evaluation in COVID-19, structured follow-up after recovery, and more consistent research methods to allow better comparisons across viral infections.

## Introduction and background

Inflammatory neuropathy was first recorded in 1976 after influenza vaccination, which led to an increase in Guillain-Barré syndrome (GBS) [1]. GBS is a serious autoimmune disease with an acute onset of neurological deterioration with decline or no deep tendon reflexes as an outcome [2]. Neurological involvement is a known consequence of viral infections, with central and peripheral syndromes that impact functional and health-system utilization, for example, dengue infection associated with headache [3] and the influenza-associated cardio-cerebrovascular outcome [4].

COVID-19 can affect every organ system, including the nervous system, but it is primarily a respiratory disease [5]. Neurological involvement in COVID-19 has been commonly reported in over one-third of all COVID patients and, in some cases, may even be more evident than respiratory symptoms. SARS-CoV-2 can affect both the central and peripheral nervous systems, leading to a wide array of neurological manifestations ranging from headache, dizziness, and loss of smell or taste to acute cerebrovascular events (stroke), seizures, altered consciousness, and GBS [6].

Neurological complications are one of the features of many viral infections, including influenza and dengue, which manifest as encephalopathy, seizures, and immune-mediated neuropathies and have been well studied. However, the emergence of SARS-CoV-2 has raised an important clinical question: whether it follows the same viral patterns or not. Unlike influenza and dengue, COVID-19 has been associated with early sensory deficits, a higher burden of cerebrovascular events, widespread neuroinflammatory injury, and persistent post-acute neurological symptoms. Understanding the underlying mechanisms, such as neuroinflammation, endothelial dysfunction, or direct viral neurotropism, can help guide targeted clinical management. Clarifying where SARS-CoV-2 fits within the viral neurocomplications is essential, as this helps influence diagnostic suspicion, acute management strategies, and the need for structured long-term neurological follow-up [6-9]. 

In light of this gap, we undertook a comprehensive systematic review to compare the neurological complications associated with COVID-19 to those associated with other viral infections, focusing on influenza, GBS, and dengue virus infection. We broadly define “neurological complications” as any clinical condition affecting the central or peripheral nervous system that arises in the context of an acute viral infection or its aftermath. Our aim is to characterize the range of COVID-19-related neurological sequelae (encompassing post-viral syndromes, as well as incidence, severity, and types) and to evaluate how these overlap with or differ from neurological complications reported with other viruses. We would also assess whether COVID-19 poses a higher or more specific neurological risk than other viral illnesses.

## Review

Methodology

Study Design

This systematic review was conducted in accordance with the Preferred Reporting Items for Systematic Reviews and Meta-Analyses (PRISMA) [10] guidelines. The protocol was prospectively registered in the International Prospective Register of Systematic Reviews (PROSPERO; ID: CRD420251064831) [11] to ensure transparency and adherence to predefined methods.

Search Strategy

A comprehensive literature search was performed across three electronic databases, which were PubMed/Medline, Scopus, and Web of Science, from January 1, 2000, to February 28, 2025, to capture studies on neurological complications of viral infections, with a focus on recent COVID-19 evidence. The search strategy combined controlled vocabulary (e.g., MeSH terms for PubMed) and free-text terms, using Boolean operators (AND, OR, NOT) to ensure broad coverage. Key search terms included: “COVID-19”, “SARS-CoV-2”, “influenza”, “dengue”, “neurological complications”, “neuroinflammation”, “anosmia”, “stroke”, “Guillain-Barré syndrome”, “encephalopathy”, “encephalitis”, “neurotropism”, and “post-viral syndromes”. Additional studies were identified through backward citation searching (snowballing) by reviewing reference lists of included articles and relevant reviews. No language restrictions were applied, and non-English articles were translated using professional services when necessary. Filters were used to exclude animal studies, editorials, and opinion pieces. The search was last updated on February 28, 2025, to ensure inclusion of the most recent evidence.

Eligibility Criteria

Inclusion criteria: Studies were included if they met the following criteria: Population: Patients with laboratory-confirmed (e.g., PCR, serology) infections of SARS-CoV-2, influenza, or dengue virus, or post-infectious GBS associated with these viruses were included. Studies involving clinically diagnosed infections were included if laboratory confirmation was not feasible (e.g., in resource-limited settings). Studies were included: (i) If exposure is acute or post-acute, neurological complications affecting the central or peripheral nervous system, including but not limited to headache, anosmia, stroke, encephalopathy, encephalitis, seizures, or GBS; (ii) If the study design is randomized controlled trials, observational studies (e.g., cohort studies, case-control studies, case series, or case reports) reporting primary data; (iii) If outcomes include incidence, prevalence, severity, or clinical characteristics of neurological complications. No geographic restrictions were applied, given the global nature of COVID-19 and influenza pandemics and the widespread prevalence of dengue and GBS.

Exclusion criteria: Studies lacking confirmed viral infections (e.g., suspected cases without diagnostic evidence, except in resource-limited settings), non-primary research (e.g., literature reviews, editorials, commentaries), and studies focusing on non-viral neurological conditions or experimental models (e.g., animal studies) were excluded**.**

Study Selection

**Figure 1:**
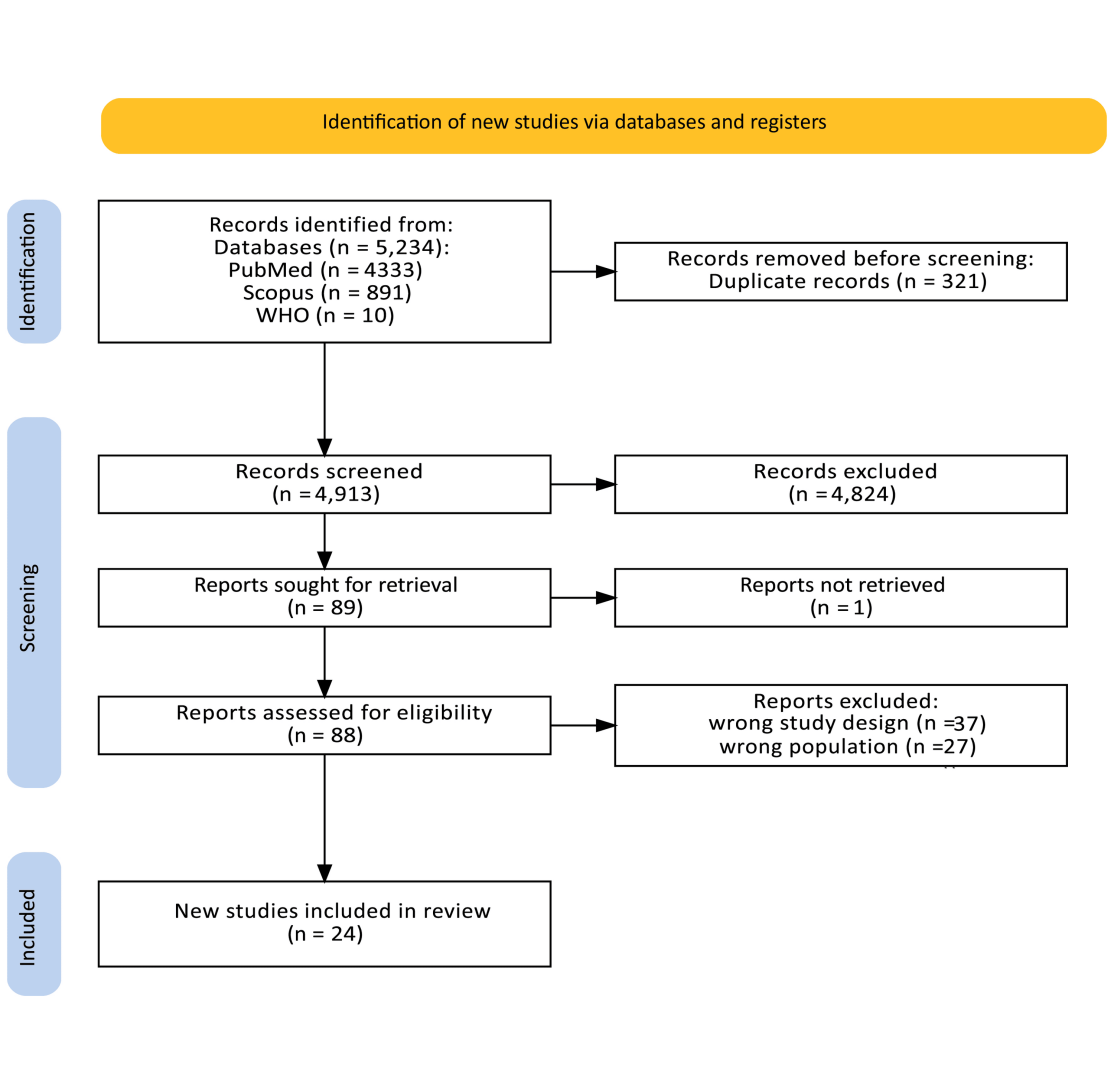
PRISMA Diagram of the Scoping Review Process PRISMA, Preferred Reporting Items for Systematic Reviews and Meta-Analysis.

The search results were imported into EndNote X9 for deduplication using an automated algorithm followed by manual verification to ensure accuracy. Two independent reviewers (ISB and CFO) screened titles and abstracts against the eligibility criteria using Rayyan, a web-based systematic review tool. Full-text articles of potentially eligible studies were retrieved and independently assessed by the same reviewers. Discrepancies were resolved through discussion, with a third reviewer (VO) consulted when consensus could not be reached. A Preferred Reporting Items for Systematic Reviews and Meta-Analyses (PRISMA) flow diagram (Figure 1) illustrates the study selection process. 

Data Extraction

Data were extracted using a standardized, pilot-tested form in Microsoft Excel. One reviewer (VO) extracted data, which were independently verified by a second reviewer (ISB) to ensure accuracy. The extracted data included the following.

Study characteristics included author, year, country, study design, and methodology. Population characteristics included sample size, age, sex, and comorbidities. Exposure and comparator data included the type of viral infection (SARS-CoV-2, influenza, dengue, or GBS-associated infections) and control groups, if applicable. Outcomes included the type, incidence, prevalence, and severity of neurological complications, including diagnostic methods such as neuroimaging and cerebrospinal fluid (CSF) analysis. Statistical data included effect sizes (e.g., odds ratios and hazard ratios), confidence intervals, and p-values. Quality assessment included risk of bias and study quality scores. In cases of missing or unclear data, corresponding authors were contacted via email for clarification, with a two-week response period.

Risk of Bias and Quality Assessment

Quality assessment revealed a moderate overall risk of bias across the included studies. Among the 11 case series and case reports evaluated using the Joanna Briggs Institute (JBI) Critical Appraisal Checklist, scores ranged from 62.5% to 75.0% (median: 75.0%), indicating predominantly moderate quality. Common limitations included incomplete follow-up data, lack of clear case definitions, and absence of statistical analysis of outcomes. The six observational studies assessed using the Newcastle-Ottawa Scale (NOS) scored between 55.6% and 77.8% (median: 66.7%), reflecting moderate risk of bias primarily due to potential selection bias, inadequate control for confounding, and incomplete outcome ascertainment. The three randomized controlled trials or clinical trials demonstrated low risk of bias (100% of domains at low risk), with adequate randomization, allocation concealment, and blinding where feasible. The self-controlled case series and population-based surveillance studies showed low to moderate risk (66.7-77.8%), with the primary concern being generalizability due to specific geographic or healthcare settings. The systematic review included in the evidence base scored 66.7% on the AMSTAR-2 tool, indicating moderate methodological quality. Notably, several early pandemic studies had methodological constraints related to rapid publication timelines, inconsistent diagnostic criteria, and variable neuroimaging protocols. These quality considerations were incorporated into the interpretation of findings, particularly when synthesizing evidence on the incidence and severity of neurological complications across viral infections.

Data Synthesis

Data were summarized using a narrative approach. We grouped studies by viral infection type and by neurological outcome. We compared study designs, populations, case definitions, diagnostic methods, and reported neurological complications. We described key findings in text and tables to highlight patterns, similarities, and differences across studies.

Ethical considerations

As this was a systematic review of published literature, no ethical approval was required. The study was conducted in accordance with the Declaration of Helsinki. All tools, 9PRISMA [10], Prospero [11], The Newcastle-Ottawa Scale [12], JBI [13] and Rayyan [14] used within this study were free to use.

Identification of relevant study data

The initial search across PubMed, Scopus, and Web of Science databases yielded 5,234 records. After removal of 321 duplicate entries, 4,913 records were retained for screening. Following title and abstract screening, 4,824 studies were excluded as they did not meet the inclusion criteria; 89 reports were sought for full-text retrieval, of which one report could not be accessed. The remaining 88 full-text articles were assessed for eligibility. During full-text evaluation, 37 articles were excluded due to inappropriate study design (such as reviews or experimental models), and twenty-seven articles were excluded for involving the wrong population (non-viral neurological conditions or unconfirmed infections).

A total of 24 studies met all inclusion criteria and were incorporated into the final systematic review. These studies encompassed diverse viral infections, primarily SARS-CoV-2, influenza virus, and dengue virus, and included post-viral GBS cohorts. The included studies varied in design, covering retrospective and prospective observational studies, case series, registry analyses, and national cohort datasets. No meta-analysis was performed due to substantial clinical and methodological heterogeneity (e.g., varying definitions of neurological complications, diverse study designs, and inconsistent reporting of outcomes), as evidenced by anticipated I² > 50% in preliminary assessments. The study selection process is illustrated in Figure 1.

Characteristics of included studies

The 24 included studies were published between 2006 and 2025, with the majority (n=18) focusing on SARS-CoV-2 (COVID-19) and published from 2020 onward. Study designs varied: 11 were case series or case reports, six were retrospective observational studies (including registry and autopsy analyses), three were randomized controlled trials or clinical trials, two were population-based surveillance studies, one was a self-controlled case series, and one was a comparative study. Sample sizes ranged from 1 (case reports) to 44,564,345 (national registry linkage study). Populations were predominantly adults (median age range: 25-77 years across studies), with mixed sex distributions (where reported, 42-76% female). Seven studies included comparators (e.g., post-vaccination vs. post-infection groups, or historical controls), while the remainder were descriptive. Neurological complications were defined variably but generally encompassed central nervous system (CNS) manifestations (e.g., encephalitis, stroke) and peripheral nervous system (PNS) manifestations (e.g., neuropathy, anosmia). Diagnostic criteria included clinical assessments, neuroimaging (e.g., MRI), CSF analysis, nerve conduction studies (NCS), and biomarkers (e.g., neurofilament light chain (NfL)) (Table 2). Risk of bias (Table 1) was moderate in most observational studies (median NOS score: 66.7%) due to potential selection bias and lack of controls; case series/reports scored moderate on the JBI checklist (median: 75.0%) owing to incomplete follow-up data. Key characteristics are summarized in Table 2.

**Table 1:** Risk of Bias and Quality Assessment of Included Studies (Percentage-Based Scoring) All scores are presented as percentages for standardized reporting. For JBI: percentage = (criteria met/total criteria) × 100. For NOS: percentage = (score/9) × 100. For RoB 2: percentage = (domains at low risk/5 domains) × 100. Classification thresholds: Low risk = ≥70% (JBI), ≥77.8% (NOS), or 100% (RoB 2); Moderate risk = 50–69% (JBI), 44.4–66.7% (NOS), or 50–79% (RoB 2); High risk = <50% (JBI), ≤33.3% (NOS), or <50% (RoB 2). No studies in this review were classified as high risk of bias. JBI: Joanna Briggs Institute; NOS: Newcastle-Ottawa Scale

Reference	Author (Year)	Study Design	Appraisal Tool	Raw Score	Percentage (%)	Risk of Bias/Quality
[1]	De Souza et al. (2022)	Case series	JBI (Case Series)	6/8	75.0	Moderate
[3]	Domingues et al. (2006)	Descriptive study	NOS	5/9	55.6	Moderate
[5]	Dias et al. (2022)	Observational study	NOS	6/9	66.7	Moderate
[7]	Abuawwad et al. (2024)	Case report	JBI (Case Report)	5/8	62.5	Moderate
[15]	García-Azorín et al. (2021)	Multicenter registry study	NOS	7/9	77.8	Low
[16]	Gelpi et al. (2023)	Postmortem case series	JBI (Case Series)	6/8	75.0	Moderate
[17]	Assiri et al. (2022)	Case series	JBI (Case Series)	6/8	75.0	Moderate
[18]	Kaya Tutar et al. (2021)	Case series	JBI (Case Series)	5/8	62.5	Moderate
[19]	Agrawal et al. (2022)	Autopsy case series	JBI (Case Series)	6/8	75.0	Moderate
[20]	Valkucakova et al. (2023)	Case presentation	JBI (Case Report)	5/8	62.5	Moderate
[21]	Gusdon et al. (2022)	Randomized controlled trial	RoB 2	5/5 domains	100.0	Low
[22]	Imam et al. (2023)	Randomized controlled trial	RoB 2	5/5 domains	100.0	Low
[23]	Stępień et al. (2023)	Comparative study	NOS	6/9	66.7	Moderate
[24]	Pires et al. (2022)	Randomized clinical trial	RoB 2	5/5 domains	100.0	Low
[25]	Avula et al. (2020)	Case report	JBI (Case Report)	5/8	62.5	Moderate
[26]	Danics et al. (2021)	Retrospective neuropathology	NOS	6/9	66.7	Moderate
[27]	Yoon et al. (2024)	Self-controlled case series	Modified NOS	6/9	66.7	Moderate
[28]	Glaser et al. (2012)	Population-based observational	NOS	7/9	77.8	Low
[29]	Ciruela et al. (2025)	Retrospective surveillance	NOS	7/9	77.8	Low
[30]	Popescu et al. (2017)	Retrospective case series	JBI (Case Series)	6/8	75.0	Moderate
[31]	Sahu et al. (2014)	Prospective cohort	NOS	6/9	66.7	Moderate
[32]	Verma et al. (2011)	Retrospective observational	NOS	6/9	66.7	Moderate
[33]	Neri et al. (2019)	Systematic review	AMSTAR-2	10/15 items	66.7	Moderate
[34]	Fung et al. (2023)	Consecutive case series	JBI (Case Series)	6/8	75.0	Moderate

**Table 2:** Characteristics of Included Studies

Reference	Author (Year)	Study Design	Sample Size (N)	Population (Age, Sex)	Virus/Exposure	Key Neurological Outcomes	
[1]	De Souza et al. (2022)	Case series	4	Adults (age not specified; all male)	ChAdOx1 nCoV-19 vaccine	Chronic inflammatory demyelinating polyneuropathy (CIDP)	
[3]	Domingues et al. (2006)	Descriptive study	Not specified	Adults (age/sex not specified)	Dengue virus (acute)	Headache (more intense in classic dengue fever)	
[5]	Dias et al. (2022)	Observational study	864 (17 with neurological manifestations)	Adults (age/sex not specified; 70.6% with comorbidities)	SARS-CoV-2 (acute)	Stroke (58.8%), paraplegia, encephalopathy; 50% mortality in stroke subgroup	
[7]	Abuawwad et al. (2024)	Case report	1	44 years (male)	Janssen COVID-19 vaccine	Guillain-Barré syndrome (GBS)	
[15]	García-Azorín et al. (2021)	Multicenter registry study	233	Mean 61.1 years (42.1% female)	SARS-CoV-2	Stroke (27%), neuromuscular symptoms (23.6%), altered mental status (23.6%), anosmia (17.6%), headache (12.9%), seizures (11.6%); persistent symptoms in 33%	
[16]	Gelpi et al. (2023)	Postmortem case series	32	Elderly (age not specified)	SARS-CoV-2 (fatal acute)	White matter damage (100%), microglial activation (100%), infarcts (22%), hypoxic-ischemic damage (40%)	
[17]	Assiri et al. (2022)	Case series	18	Adults (predominantly older males for stroke)	COVID-19 vaccines (Pfizer/AstraZeneca)	Cerebral venous thrombosis (16.7%), ischemic stroke (44.4%), seizures (16.7%), optic neuritis (11.1%), GBS (5.6%), Miller Fisher syndrome (5.6%)	
[18]	Kaya Tutar et al. (2021)	Case series	3	Adults (age/sex not specified)	SARS-CoV-2	Inflammatory/demyelinating CNS lesions (e.g., optic neuritis, multifocal plaques)	
[19]	Agrawal et al. (2022)	Autopsy case series	20	Mean 66.2 years (70% male)	SARS-CoV-2 (fatal acute)	Vascular pathologies (80%), microglial activation (80%), encephalitis-like changes (30%)	
[20]	Valkucakova et al. (2023)	Case presentation	2	Adults (females)	SARS-CoV-2	Catatonia (secondary to neuroinflammation)	
[21]	Gusdon et al. (2022)	Randomized controlled trial	24	Adults (severe COVID-19)	SARS-CoV-2 (acute); OP-101 treatment	Elevated NfL, GFAP, tau (neuronal injury markers); reduced in treatment groups	
[22]	Imam et al. (2023)	Randomized controlled trial	66	Adults (1:1 allocation)	SARS-CoV-2 (post-infectious)	Anosmia (improved in DTPA group)	
[23]	Stępień et al. (2023)	Comparative study	45 (study) + controls	Adults (post-COVID survivors)	SARS-CoV-2 (post-infectious)	Peripheral neuropathy (reduced NCS parameters, e.g., sural nerve amplitude)	
[24]	Pires et al. (2022)	Randomized clinical trial	80	Adults (outpatients)	SARS-CoV-2 (post-infectious)	Olfactory dysfunction (anosmia/hyposmia; improved with training)	
[25]	Avula et al. (2020)	Case report	1	25 years (sex not specified)	SARS-CoV-2 (acute)	Locked-in syndrome (secondary to pontine strokes)	
[26]	Danics et al. (2021)	Retrospective neuropathology	36 (13 HSV, 23 neurosyphilis)	Mean 53–60 years	HSV/neurosyphilis (comparators)	Tau pathology (61.5–82.6%), amyloid-β plaques (30.8–47.8%)	
[27]	Yoon et al. (2024)	Self-controlled case series	44,564,345	>18 years (sex not specified)	COVID-19 vaccines	Facial palsy (increased risk in 1–28 days post-vaccination)	
[28]	Glaser et al. (2012)	Population-based observational	2,069 (77 with complications)	Median 9 years (58% male)	Influenza A(H1N1)pdm09	Encephalopathy/encephalitis (37.7%), seizures (57.1%), meningitis (3.9%), GBS (1.3%); 4% incidence	
[29]	Ciruela et al. (2025)	Retrospective surveillance	5,931 (9 with complications)	Median 62 years (sex not specified)	Influenza A/B	Encephalitis (66.7%), GBS (11.1%), meningitis (11.1%), myelitis (11.1%); 1.5 per 1,000 incidence	
[30]	Popescu et al. (2017)	Retrospective case series	32 (7 with complications)	Median 31 years (85.7% female)	Influenza B	Encephalitis (85.7%), cerebellar ataxia (14.3%)	
[31]	Sahu et al. (2014)	Prospective cohort	Not in extract*	Adults/children (age/sex not specified)	Dengue virus	Encephalitis, GBS, myelitis (prevalence 6–12%)	
[32]	Verma et al. (2011)	Retrospective observational	26	Mean 29.1 years (69.2% male)	Dengue virus	Encephalitis/encephalopathy (15.4%), myelitis (3.8%), myositis (7.7%), GBS (11.5%), brachial neuritis (38.5%)	
[33]	Neri et al. (2019)	Systematic review	47 studies	Mixed (74.4% Asia)	Dengue virus	Encephalic (40.4%), peripheral (29.7%), demyelinating (17%), spinal (12.7%); low mortality	
[34]	Fung et al. (2023)	Consecutive case series	21	Mean 50.7 years (76% female)	SARS-CoV-2 (post-infection/vaccination)	Functional movement disorders (43%; higher post-vaccination)	

Neurological complications by virus

SARS-CoV-2 (COVID-19)

Eighteen studies reported neurological complications associated with SARS-CoV-2, encompassing acute infection (n=10), post-infectious (n=5), and vaccine-related (n=3) contexts. Incidence varied: 1.96% in hospitalized cohorts [5] to 27-58.8% for specific manifestations in symptomatic subgroups [5,15]. Prevalence of any neurological complication was 33-43% in registry/outpatient data [15]. Common CNS manifestations included stroke (27-58.8%; ischemic predominant) [5,15,17,25], encephalopathy/encephalitis (15.4-66.7%) [5,16,18,19], seizures (16.6-16.7%) [5,15,17], and white matter/vascular pathologies (80-100% in autopsies) [16,19]. PNS manifestations featured anosmia/olfactory dysfunction (17.6-100% in affected subgroups) [15,22,24], neuropathy (including demyelination; reduced NCS in 100% of tested survivors) [18,23], GBS (5.6-11.5%) [5,7,17], and facial palsy (<0.1% post-vaccination) [27]. Rare findings included catatonia [20], locked-in syndrome [25], chronic inflammatory demyelinating polyneuropathy (CIDP) [1], functional motor disorders (43%) [24], and biomarkers of neuronal injury (elevated NfL/GFAP in 100%) [21]. Mortality was high in severe cases (50% in stroke subgroups) [5]; persistent symptoms occurred in 33% [15]. Vaccine-related complications were rare (<0.1%) [17,27].

Influenza Viruses

Four studies addressed influenza A/B complications [28-30], with an incidence of 1.2-1.5 per 1,000 severe cases [28,29]. In 8,032 cases, 93 (1.2%) had neurological manifestations [28-30]. CNS issues predominated: encephalopathy/encephalitis (37.7-85.7%) [28-30], seizures (57.1%) [28], meningitis (3.9-11.1%) [28-30], and cerebellar ataxia (14.3%) [30]. PNS included GBS (1.3-11.1%) [28,30] and myelitis (11.1%) [29]. No mortality or sequelae was in some cohorts [29]; mean hospital stay was 25.6 days [29]. Across the included studies, the most consistently reported manifestations were encephalitis/encephalopathy and seizures [28,30]. A 10-year population-based surveillance study in California found that 4% of severe or fatal H1N1 cases had primary neurologic complications, with encephalopathy/encephalitis (29 cases) and seizures (44 cases) being most common [28]. Another decade-long surveillance study from Catalonia identified encephalitis in 66.7% of severe influenza cases with neurological involvement [29]. Other reported complications included GBS, meningitis, and myelitis [28-30].

Dengue Virus

Three studies reported dengue complications [31-33], with a prevalence of 6-12% [31-33]. Headache was universal but more intense in classic vs. hemorrhagic fever [3], CNS: encephalopathy/encephalitis (15.4-40.4%) [31-33], PNS/spinal: GBS (11.5-57.1%), myelitis (3.8-83.3%), brachial neuritis (38.5%), and myositis (7.7%) [31-33]. Low mortality (<5%) was reported; most recovered with supportive care [31-33] (Table 3). Dengue virus infection was associated with a broad range of neurological complications, stemming from direct neurotropism, immune-mediated phenomena, and systemic metabolic disturbances [32,33]. The most common manifestations reported were encephalitis/encephalopathy (15.3% in one cohort) and GBS [32,33]. Other significant complications included myelitis, myositis, and peripheral syndromes such as brachial neuritis (neuralgic amyotrophy), which affected 38% of patients in one tertiary center cohort [32]. Despite the potential for severe neurological involvement, studies indicated that most patients recovered well with supportive care, and mortality remained low [33].

**Table 3:** Summary of Neurological Complications by Virus

Virus	Common CNS Manifestations (Prevalence/Incidence)	Common PNS/Other Manifestations (Prevalence/Incidence)	Mortality/Sequelae
SARS-CoV-2	Stroke (27–58.8%), encephalopathy/encephalitis (15.4–66.7%), seizures (11.6–16.7%), white matter damage (80–100%) [5,15-19,25]	Anosmia/olfactory dysfunction (17.6–100%), neuropathy/demyelination (100% in tested), GBS (5.6–11.5%), facial palsy (<0.1%), functional disorders (43%), CIDP (100% in series), catatonia (100% in series), locked-in syndrome (case) [1,5,7,15,17,18,20,22–25,27,34]	50% in stroke; persistent symptoms 33% [5,15]
Influenza	Encephalopathy/encephalitis (37.7–85.7%), seizures (57.1%), meningitis (3.9–11.1%) [28–30]	GBS (1.3–11.1%), myelitis (11.1%), cerebellar ataxia (14.3%) [28–30]	Low (0–<5%); no sequelae in some [29]
Dengue	Encephalopathy/encephalitis (15.4–40.4%), headache (universal) [3,31-33]	GBS (11.5–57.1%), myelitis (3.8–83.3%), brachial neuritis (38.5%), myositis (7.7%) [31-33]	Low (<5%); good recovery [31-33]

Neurological complications associated with SARS-CoV-2

The included studies reported a wide spectrum of neurological complications associated with COVID-19, affecting both the central and peripheral nervous systems.

CNS Manifestations

Cerebrovascular events: Acute stroke was a prominent and severe complication. A registry study from Spain identified stroke in 27% of patients with neurological presentations [15]. In a hospital-based cohort, stroke was the most common neurological manifestation, occurring in 10 of 17 patients (58.8%) and was associated with a high mortality rate of 50% [5]. Other studies also reported cases of ischemic stroke and intracranial hemorrhage [17].

Encephalopathy and encephalitis: Altered mental status and encephalopathy were frequently observed, reported in 23.6% of cases in the Spanish registry [15]. Postmortem neuropathological studies provided significant insights, revealing diffuse white matter damage, extensive microglial activation (80%), and inflammatory changes in all fatal cases examined, often concentrated in the brainstem [16,19]. Notably, these studies did not detect viral RNA in brain tissue, suggesting that CNS injury is likely secondary to systemic inflammation and hypoxia rather than direct viral invasion [19]. One study also documented a case of acute hemorrhagic leukoencephalitis [19].

**Figure 2:**
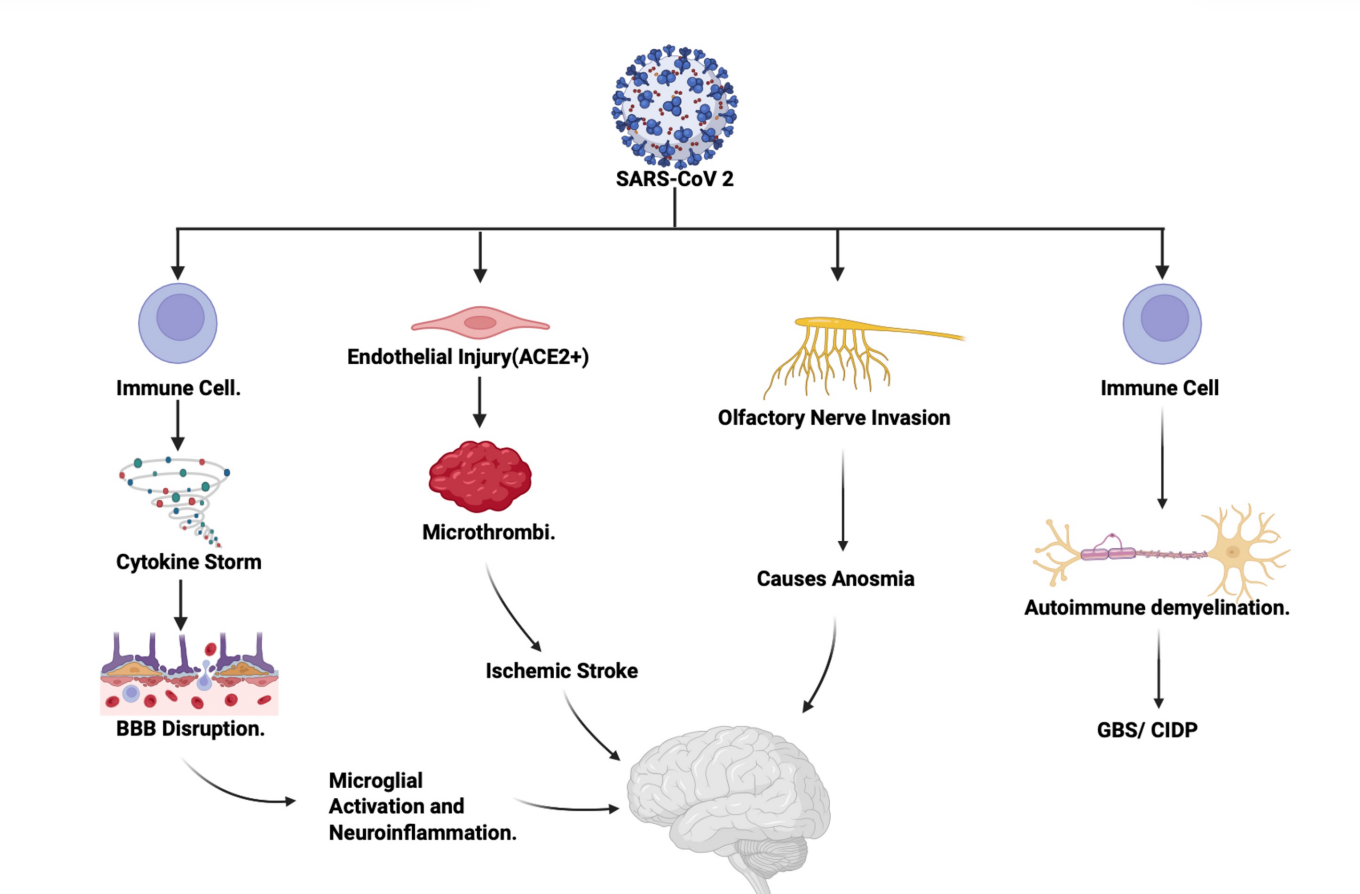
COVID-19 Neuroinvasion Pathways Mechanisms of SARS-CoV-2–associated neurological injury. This schematic was created by the authors for this study to summarize reported mechanisms including immune-mediated neuroinflammation, endothelial injury, olfactory nerve involvement, and autoimmune demyelination. Figure created by the authors using BioRender.com.

Seizures and other CNS syndromes: Seizures were reported in 11.6% of patients with neurological symptoms [15], with other studies corroborating this finding [5,17]. Rarer but severe syndromes were also documented, including cases of COVID-19-associated catatonia, proposed to be triggered by neuroinflammation [20], and a case of locked-in syndrome secondary to pontine strokes in a young patient [25]. A conceptual overview of the major pathophysiologic mechanisms implicated in SARS-CoV-2 neuroinvasion, including cytokine-mediated disruption, endothelial injury with microthrombi formation, direct olfactory neuronal invasion, and autoimmune demyelination, is presented in Figure 2.

PNS and Sensory Manifestations

Anosmia and olfactory dysfunction: Anosmia was a hallmark symptom, reported in 17.6% of patients in a large registry [15]. Its significance was further highlighted by clinical trials focused on treating persistent post-COVID olfactory dysfunction, confirming its high prevalence [22,24].

Demyelinating conditions and GBS: A spectrum of autoimmune demyelinating conditions was associated with SARS-CoV-2 infection [18] and vaccination [1]. GBS was identified as a complication of both the infection itself [5] and, rarely, post-vaccination [7]. In a case series, four patients developed CIDP within weeks of vaccination [1].

Peripheral neuropathy and motor disorders: Evidence of peripheral nerve damage was confirmed in COVID-19 survivors. A comparative study using nerve conduction tests found significant abnormalities indicative of axonal injury and secondary demyelination persisting months after recovery [23]. Furthermore, functional motor disorders, including tremor and gait dysfunction, were identified in 43% of patients referred to a specialized clinic for post-COVID or post-vaccination motor symptoms [34]. Facial palsy was also reported as a rare complication, with a study showing a slightly increased risk within 28 days post-vaccination [27].

CIDP was found in all four male patients, just 3-4 weeks after their vaccination [1]. Acute stroke was the major complication found in 58.8% of patients who had neurological complications after COVID according to the study by Dias et al. [5]. This led to 50% mortality. Other complications identified include encephalitis, encephalopathy, GBS and seizures [5,15-17]. Similarly, GBS was found in patients with COVID-19 [7]. In the series by Kaya Tutar et al. [18], three patients with SARS-CoV-2 infection developed distinct demyelinating manifestations. Case 1 showed acute inflammatory demyelination with bilateral centrum semiovale lesions. Case 2 presented optic neuritis and multifocal demyelinating plaques, meeting criteria for multiple sclerosis. Case 3 exhibited transient cerebellar and corpus callosal demyelination that resolved spontaneously. MRI findings revealed T2-FLAIR hyperintensities and variable contrast enhancement, while CSF analysis confirmed inflammatory activity. Collectively, these cases support a COVID-19-related autoimmune demyelinating spectrum. An autopsy study involving 20 critically ill COVID-19 patients (mean age 77 years) found that neuroinflammatory changes such as microglial activation (80%), microglial nodules (30%), and neuronophagia (30%) were frequent, predominantly in the brainstem. Some findings included acute hemorrhagic leukoencephalitis (5%) and vasculitis-like lesions (5%). The study did not detect any viral Rn in the brain tissue, but showed that covid 19 increases secondary vascular and inflammatory brain injury, indicating a neurological trigger for elderly patients [19].

This case series studied two women who received no COVID-19 vaccines; they presented with catatonia symptoms after neurological work-up ruled out encephalitis or structural lesions in the brain stem. Also findings like elevated IL-6, systemic inflammation, anxiety, and immune dysregulation were ruled out, which suggests that it is indirect CNS instead of viral neuroinvasion. The study concludes that COVID-19 may encourage secondary catatonia through neuroinflammation [20]. Markers like NfL, Glial fibrillary acidic protein (GFAP and Total Tau protein were confirmed to be increased in 24 patients hospitalized after COVID-19; these markers indicate axonal injury, astrocyte injury and neuronal degeneration [21]. Anosmia is the neurological complication observed in the patients of this study [22], while the COVID-19 survivors showed level of clear signals of peripheral nerve damage, which are axon injury and secondary demyelination, months even after recovery [18]. Presenting as tremor, tics, gait dysfunction or weakness, the study followed 21 patients to know the onset of these neurological findings, whether it was during the infection or after the vaccination, which confirmed functional movement disorders in 43%.

Although this study focused on the treatment of patients who had olfactory dysfunctions (anosmia and hyposmia) after COVID-19, however it still confirmed the presence of these neurological complications after the infection [24]. Meanwhile, this case study presented with locked-in syndrome [25]. The observations provide insights into the deposition of neurodegenerative proteins in neuroinfections, which might have implications for COVID-19 patients with chronic and/or post-infectious neurological symptoms and encephalitis [26]. Less than 0.1 % of the population with COVID presented with facial palsy [27].

**Figure 3:**
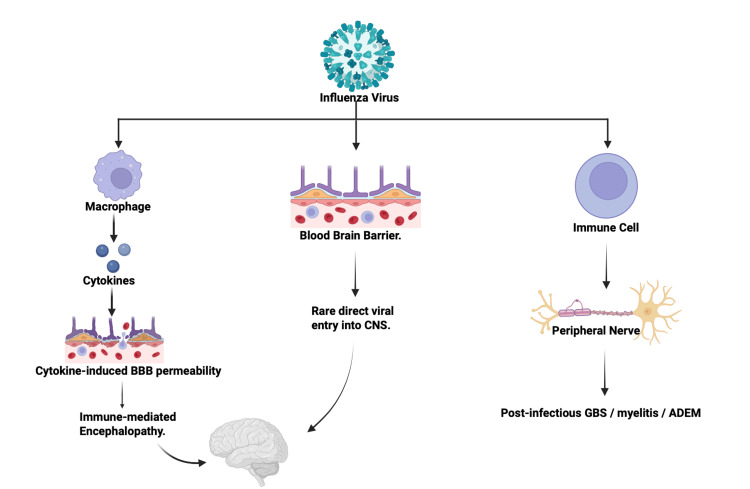
Influenza Neuroinvasion pathways Mechanisms of influenza virus–associated neurological injury. Schematic illustrating cytokine-driven immune activation, increased blood–brain barrier permeability, immune-mediated encephalopathy, rare direct central nervous system invasion, and post-infectious peripheral nervous system complications including Guillain–Barré syndrome, myelitis, and acute disseminated encephalomyelitis. Figure created by the authors using BioRender.com.

**Figure 4:**
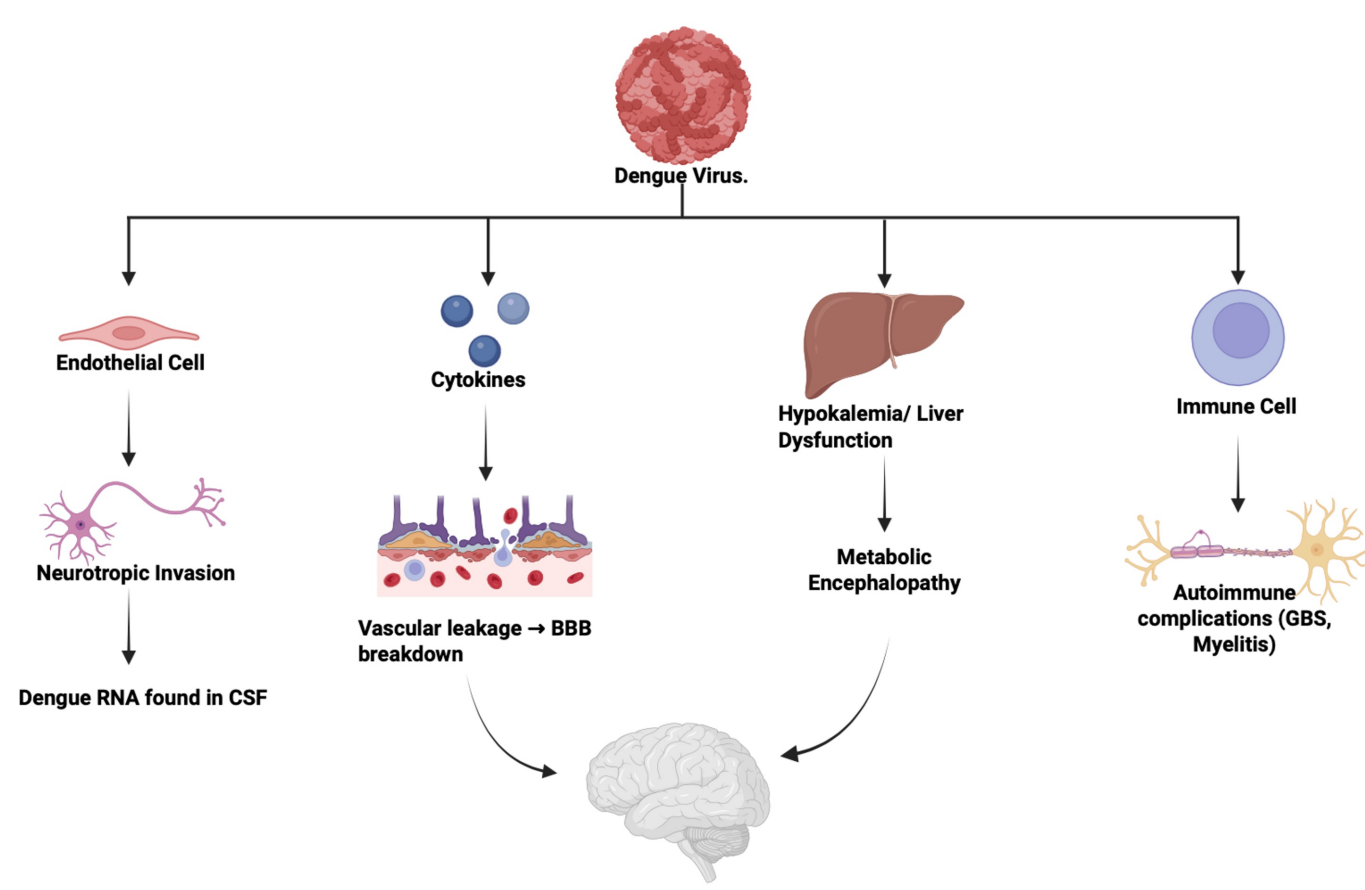
Dengue Neuroinvasion Pathways Mechanisms of dengue virus–associated neurological injury. Schematic showing direct neurotropic invasion with viral RNA detected in cerebrospinal fluid, cytokine-mediated vascular leakage and blood–brain barrier disruption, metabolic encephalopathy related to hypokalemia and liver dysfunction, and immune-mediated complications such as Guillain–Barré syndrome and myelitis. Figure created by the authors using BioRender.com.

While the neurological complications for influenza are not as elaborate as COVID-19, some major risks were still identified, especially in both the central and peripheral nervous systems. Encephalitis/encephalopathy, followed by seizures, meningitis, GBS, myelitis, and rare ataxia, were present together with both types of influenza. Among 8,032 patients with influenza, 93 individuals, representing 1.2%, developed neurological complications. Of these neurological cases, encephalitis or encephalopathy accounted for approximately 60 to 70% [28-30]. Similarly to the complications with influenza, very few differences in the proposed neuroimmune and neuroinflammatory mechanisms underlying influenza-associated neurological disease, as seen in Figure 3. Dengue viral infection presented with encephalitis, GBS, myelitis, brachial neuritis (neuralgic amyotrophy) and myositis [31-33]. It has a prevalence score of 6-12% across the studies for dengue fever; this evidence expresses around three mechanistic axes, direct neurotropism of DENV (confirmed CSF RNA), immune-mediated responses (ADEM, GBS), and metabolic/systemic effects (hypokalemia and liver dysfunction) [31-33]. Despite significant neurologic involvement, most patients recovered well with supportive care, and mortality remained low (<5%). The multifactorial pathways through which dengue virus produces neurological injury, including direct neurotropic invasion, immune-mediated demyelinating complications, and metabolic encephalopathy, are summarized in Figure 4.

Discussion

The neurological complications of COVID-19 have become a major worry, with a range of symptoms that are different from those seen in other viral infections in terms of how common and serious they are. The main results of recent systematic reviews and meta-analyses indicate that neurological symptoms are common and varied in COVID-19, with some syndromes occurring more frequently than in other viral illnesses [35-38].

Principal Findings

Recent studies have consistently shown that COVID-19 is linked to a much wider and more serious range of neurological problems than other viral infections like influenza, SARS, and MERS. Numerous studies have recorded that a significant percentage of COVID-19 patients exhibit neurological symptoms at various stages of their illness. For example, a study found that 36.4% of COVID-19 patients in Wuhan showed neurological signs, which is much higher than what was seen in other coronavirus outbreaks and respiratory viruses [39]. Unlike other viral infections that typically present with nonspecific neurological symptoms such as headache or mild dizziness, COVID-19 exhibits a distinct propensity for involvement of both the central and peripheral nervous systems. During the acute phase, prevalent symptoms encompass headache, dizziness, and altered mental status; nevertheless, more severe complications, including ischemic stroke, encephalopathy, and GBS, have also been frequently observed [39,40]. COVID-19 is linked to a wide array of neurological complications, affecting both the central and peripheral nervous systems. The most common neurological symptoms in COVID-19 are headache, altered mental status, myalgia, anosmia (loss of smell), and dysgeusia (loss of taste) [35-37].

About one-third of patients report these symptoms, and the pooled prevalence estimates for myalgia, anosmia, and dysgeusia are each around 20-40% [35-37]. COVID-19 seems to cause a unique syndrome that is marked by strong smell and taste problems. Anosmia (loss of smell) and ageusia (loss of taste) have been reported at very high rates, even in cases where respiratory symptoms are minimal or absent; these sensory deficits may precede the onset of more classical respiratory manifestations [41,42]. This pattern sharply contrasts with other respiratory viruses, where early sensory changes are either significantly less common or not a salient characteristic. The high frequency of these symptoms facilitates early clinical recognition and indicates a potential neuroinvasive mechanism specific to SARS-CoV-2 [39]. Anosmia and dysgeusia are especially noticeable in COVID-19 compared to other respiratory viral infections, like the flu, where these symptoms are less common and less severe [38,41].

Cerebrovascular accidents, particularly ischemic stroke, have become a prominent neurological complication associated with COVID-19. Research has shown that COVID-19 patients are several times more likely to have an ischemic stroke than people with the flu. Hazard ratios show that this risk is significantly higher [40,43]. Stroke is the most common serious neurological diagnosis, with a combined prevalence of about 2% among COVID-19 patients who are hospitalized [36,38].

This rate is higher than what is seen in other viral infections, such as the flu and previous coronavirus outbreaks (SARS, MERS), where stroke is not a common problem [38]. The fundamental factors contributing to this observation are the hyperinflammatory state and a prothrombotic environment induced by SARS-CoV-2 infection. These effects are exacerbated by endothelial dysfunction and a severe cytokine storm, which seem to promote thromboembolic events not typically observed in other viral infections [43,44]. The heightened risk of stroke in COVID-19 is believed to be associated with a pro-thrombotic state and an exaggerated inflammatory response, as indicated by elevated markers such as D-dimer and C-reactive protein [38].

Along with stroke, there are many reports of encephalopathy and changes in consciousness in people with COVID-19. Severe cases, especially those in intensive care units, have exhibited elevated rates of encephalopathy, delirium, and other cognitive disturbances, a trend associated with both disease severity and systemic hypoxia. Encephalopathy and acute confusion/delirium are prevalent, particularly among older adults, with a prevalence reaching 34% in individuals aged ≥60 years [36]. Encephalopathy can manifest in various severe viral infections; however, its incidence and severity in COVID-19, especially in the context of critical illness and hypoxia, seem to be elevated [38].

There have also been reports of more serious neurological syndromes, such as stroke, encephalopathy, encephalitis, acute disseminated encephalomyelitis (ADEM), and GBS. Additionally, reports of GBS linked to COVID-19 underscore an increased risk for immune-mediated polyneuropathies; certain studies indicate that the likelihood of developing GBS in the context of COVID-19 is markedly elevated compared to non-COVID-19 populations [43]. Even though GBS is less common than some other neurological symptoms, its higher-than-expected rate shows that SARS-CoV-2 can cause immune responses that damage peripheral nerves. Peripheral nervous system complications, such as GBS, have been reported but remain relatively rare (1%) [35,38,45,46]. Moreover, emerging data on encephalitis, a complication with a pooled incidence of approximately 0.215% overall and rising to nearly 6.7% among critically ill patients, further underscores the need for prompt and precise neurodiagnostic evaluations [47].

The high mortality rate associated with encephalitis and its potential to cause permanent neurological damage mandates that clinicians maintain a high index of suspicion for such complications, especially in patients presenting with altered mental states, seizures, or rapid clinical deterioration. These findings reinforce our overall conclusion that COVID-19 imposes a unique burden on neurological health that clearly differentiates it from other viral infections and calls for integrated, multidisciplinary approaches to both acute management and long-term rehabilitation [39]. In pediatric populations, abnormal neuroimaging findings are present in a substantial proportion of children with neurological symptoms, including neurovascular events, ADEM-like lesions, encephalitic patterns, myelitis, and transient splenial lesions [48]. The spectrum of findings in children underscores the need for vigilance, as some patterns (e.g., transient splenial lesions) are less commonly reported in other viral infections [48].

Beyond the individual neurological syndromes, another important distinction is the temporal pattern of neurological involvement. Several studies suggest that in patients with COVID-19, neurological symptoms may occur at any stage of the illness; they might present before, simultaneously with, or after the onset of respiratory illness [39,49]. This stands in contrast to the more predictable temporal evolution seen in other viral infections, where neurological complications typically follow the peak of systemic illness. Data also indicate that a significant number of patients, especially those with severe illness, continue to experience neurological deficits well into the recovery phase, a phenomenon often described as part of "long COVID" [50,51]. Overall, these principal findings affirm that while some overlap exists with neurological presentations of other viral infections, COVID-19 exhibits a distinctive profile characterized by higher incidence rates, a broader range of neurological symptoms, and a tendency for severe complications such as stroke and encephalopathy.

Strengths and Limitations of the Selected Studies

A substantial strength of the research supporting these conclusions is the diversity of methodological approaches adopted. The corpus of evidence includes systematic reviews, meta-analyses, large cohort studies, and detailed case stories from both single-center and multicenter research. Much of this research is obtained from high-quality, peer-reviewed journals and reflects robust statistical analyses that give a precise quantification of the numerous neurological symptoms of COVID-19 [39,40,43]. The merits of the present literature include the high sample sizes and methodical methodologies utilized in meta-analyses, which provide robust prevalence estimates for neurological problems [35-37]. Notably, some investigations have high sample sizes, often exceeding tens of thousands of patients, which strengthens the reliability of the prevalence estimates and facilitates relevant comparisons with other viral diseases. The inclusion of both adult and pediatric populations allows for a thorough assessment across age groups [48]. Many studies have adopted standardized diagnostic criteria and have attempted to separate direct viral effects from secondary consequences of critical disease [38].

The inclusion of detailed neurological examinations paired with neuroimaging, CSF analysis, and assessments of inflammatory biomarkers (such as IL-6, IL-8, and MCP-1) has further strengthened these studies by providing insights into the potential pathophysiologic mechanisms underlying neurological damage [39,52]. Such mechanistic findings are crucial in differentiating whether brain harm originates from direct viral invasion, systemic inflammatory responses, or a combination of both.

Despite these strengths, numerous limitations are obvious across the literature. A fundamental problem is the pronounced variability in study design, sample demographics, and diagnostic criteria employed to identify neurological sequelae. Many of the studies are retrospective in nature, which renders them prone to intrinsic flaws such as selection bias, recollection bias, and poor case ascertainment [40,53]. Most studies are observational, with inherent concerns of bias and confounding [36,37]. There is great variation in study design, patient demographics, and classifications of neurological disorders, which complicates direct comparisons and pooled analysis [35,37]. For example, the wide variation in reported neurological symptom prevalence, with some studies citing as high as 88% of patients exhibiting neurological manifestations [40] and others reporting much lower frequencies, highlights the difficulty in achieving standardized measurements across disparate settings.

Additionally, discrepancies in the diagnostic methods deployed (ranging from clinical neurological tests to advanced neuroimaging and CSF analysis) have led to inconsistencies in the classification and reporting of neurological disorders. Such inconsistencies hinder the capacity to correctly compare COVID-19-related neurological consequences with those resulting from other viral infections [54]. In certain situations, the overlapping nature of symptoms such as headache, malaise, and fatigue can result in underreporting or misclassification, particularly in overwhelmed healthcare systems during peak infection periods [55,56]. Many reports lack extensive clinical, radiographic, and laboratory data, limiting the capacity to establish causation and mechanistic insights [38]. The early phase of the pandemic was typified by quick publication and inadequate peer review, significantly raising the possibility of bias [37,57]. Pediatric results remain restricted, with small sample numbers and short follow-up.

Furthermore, some data arise from questionable sources or predatory journals, which require cautious interpretation of their conclusions [58,59]. Differing healthcare practices across geographic locations and patient demographics further limit the generalizability of these conclusions. Finally, the lack of consistent follow-up lengths in many studies hinders the ability to distinguish between acute neurological signs and long-term effects, underscoring an important area that deserves further research [60,61].

Surveillance and Clinical Follow-Up Strategies

Given the diverse and often severe spectrum of neurological involvement in COVID-19, it is imperative that health systems adapt their surveillance and clinical follow-up strategies to comprehensively address these complications. First and foremost, routine neurological assessments should be integrated into the standard clinical evaluation of COVID-19 patients. This approach entails not only a thorough neuro-ophthalmologic and neurocognitive examination but also the use of standardized symptom checklists and scales to capture subtle changes such as loss of smell, taste disturbances, and the onset of headache or dizziness [39,41].

The implementation of systematic screening protocols is particularly important in emergency departments and hospital wards where patients may present with neurological deficits before respiratory symptoms become prominent. Early recognition of these signs can facilitate prompt diagnostic workups, including neuroimaging, laboratory analyses of inflammatory markers, and CSF studies, which, in turn, may accelerate the initiation of targeted interventions [52].

In the inpatient setting, particularly among those with severe disease, continuous monitoring is recommended. Neurological assessments should be performed regularly throughout the hospital stay, with immediate attention given to signs of altered consciousness, seizures, or focal neurological deficits that may indicate cerebrovascular events or encephalopathy [52]. For conditions like ischemic stroke, prompt identification is critical, and early diagnostic imaging (e.g., CT or MRI of the brain) should be used to guide urgent management [40,43].

Beyond acute care, long-term follow-up is essential, especially in light of emerging evidence on post-COVID neurological complications. "Long COVID" frequently manifests with persistent cognitive dysfunction (often described as "brain fog"), chronic fatigue, sleep disturbances, and even neuropsychiatric symptoms such as anxiety and depression [50,51]. Establishing dedicated post-COVID clinics that incorporate multidisciplinary teams comprising neurologists, psychiatrists, rehabilitation specialists, and primary care providers will be pivotal in managing these complex presentations [39,49]. Such clinics should employ standardized protocols for neuropsychological testing, neuroimaging follow-ups, and even advanced diagnostic modalities like quantitative EEG or functional MRI when indicated, to monitor the evolution or resolution of neurological abnormalities over time.

Telemedicine emerges as an especially valuable tool in this context. Remote neurological consultations allow clinicians to monitor patients who are recovering at home, ensuring continuous assessment of subtle neurological changes without requiring frequent in-person visits. This is particularly beneficial for patients residing in remote areas or those with mobility challenges due to post-COVID conditions [42,49].

Furthermore, the creation of centralized registries or electronic databases dedicated to tracking neurological outcomes in COVID-19 patients would enhance epidemiologic surveillance. These registries should compile comprehensive data covering patient demographics, clinical presentations, diagnostic results, treatment responses, and long-term outcomes, which can be used to refine predictive models and inform public health strategies [60,61]. Integration of such data with national and international health information systems is warranted to ensure rapid dissemination of findings and to support collaborative research initiatives.

Research Gaps

Although the current body of literature provides substantial evidence regarding the neurological complications of COVID-19, several important research gaps remain that must be addressed to enhance our understanding and improve patient management. One of the foremost gaps is a detailed elucidation of the pathophysiological mechanisms underlying these neurological complications. While it is generally accepted that factors such as direct viral neuroinvasion, the "cytokine storm," and endothelial dysfunction contribute to neural injury, the relative contribution of each mechanism remains unclear. In vitro and animal studies, as well as advanced molecular investigations, are needed to characterize the exact pathways through which SARS-CoV-2 invades the central nervous system and induces inflammatory cascades [39,52].

Another critical area for research is the relatively limited longitudinal data on long-term neurological outcomes following COVID-19. Although many patients exhibit persistent symptoms months after the resolution of acute infection ranging from chronic cognitive deficits and persistent headaches to more debilitating disorders such as post-encephalitic syndromes, the natural history of these conditions remains poorly defined [50,51]. Longitudinal cohort studies with robust follow-up protocols are necessary to chart the trajectory of neurological recovery, determine the incidence of permanent deficits, and identify risk factors that predispose patients to prolonged or progressive neurological deterioration [39,61].

The heterogeneity in diagnostic criteria across studies further underscores the need for standardization. Future research should adopt uniform definitions and validated scales for measuring neurological outcomes, thereby reducing the inter-study variability that currently complicates meta-analyses. Such standardization would facilitate more precise comparisons between COVID-19 and other viral infections, as well as improve our understanding of the true prevalence and severity of each neurological complication [40,53].

There is also a notable lack of data from certain geographic regions and populations. Many of the studies reviewed were conducted in high-income countries, leaving a significant gap in our understanding of how COVID-19 impacts neurological health in low- and middle-income countries. Differences in healthcare infrastructure, demographic profiles, and even viral strains might influence both the incidence and outcome of neurological complications, making it imperative that future research encompass a broader range of settings [39,40].

Pediatric populations represent another under-researched area. While most of the literature has focused on adult patients, children and adolescents may also experience distinct neurological complications that differ in presentation, severity, and recovery trajectory. Focused studies in pediatric cohorts will be essential to tailor age-specific management strategies and preventive measures [39,49].

Furthermore, although several studies have documented a correlation between severe COVID-19 and a heightened risk for neurological complications, there is insufficient data on the outcomes of mild cases or asymptomatic individuals who develop neurological symptoms. Given that some patients can present with neurological deficits in the absence of significant respiratory symptoms, understanding the full clinical spectrum across disease severities is critical [39,49].

Despite the strengths of the current literature characterized by large sample sizes, robust statistical methodologies, and multi-modal diagnostic approaches a few limitations persist. Differences in study design, variability in diagnostic criteria, and incomplete longitudinal data all contribute to challenges in accurately quantifying the true extent of neurological involvement in COVID-19. These limitations underscore the urgent need for standardized diagnostic protocols, uniform reporting methodologies, and prospective, multicenter studies that can reliably capture both the acute and long-term neurological sequelae of SARS-CoV-2 infection.

Lastly, while advanced imaging and CSF analyses have provided valuable insight into the acute neurological effects of COVID-19, the potential role of persistent viral remnants in the nervous system remains unexplored. Preliminary findings suggest the presence of inactive SARS-CoV-2 remnants in tissue samples of patients with long COVID, but the clinical significance of these findings and their relationship to ongoing neurological dysfunction deserve further investigation [47]. Collaborative, multicenter studies employing state-of-the-art neuroimaging, molecular diagnostics, and longitudinal patient monitoring will be central to closing these critical research gaps.

## Conclusions

Our comprehensive analysis reveals that COVID-19 is markedly distinct from other viral infections in terms of its neurological impact. The available evidence unequivocally supports a higher incidence and a broader spectrum of neurological complications in COVID-19, ranging from nonspecific symptoms such as headache, dizziness, and fatigue to more severe syndromes including ischemic stroke, encephalopathy, and GBS. Furthermore, the prominence of early sensory deficits, specifically anosmia and ageusia, in COVID-19 patients not only facilitates timely diagnosis but also underscores the unique neurotropic capabilities of SARS-CoV-2. More concerning is the potential for severe complications to precipitate long-term morbidity, as evidenced by the persistent neurocognitive deficits and other post-infectious syndromes observed in some patients.

From a public health perspective, the high prevalence and potential severity of neurological complications call for immediate action in both clinical practice and health system surveillance. Routine and early neurological assessments should be incorporated into the standard care pathway for COVID-19 patients, with particular attention paid to the early detection of sensory deficits, altered consciousness, and cerebrovascular events. Equally important is the establishment of post-COVID follow-up programs that leverage multidisciplinary teams and telemedicine to ensure that patients suffering from persistent or late-onset neurological complications receive appropriate evaluation and treatment. The development of centralized neurological registries will also play a critical role in monitoring long-term trends and informing targeted interventions.
